# Sustainable Power Generation Through Solar‐Driven Integration of Brayton and Transcritical CO_2_ Cycles: A Comprehensive 3E (Energy, Exergy, and Exergoenvironmental) Evaluation

**DOI:** 10.1002/gch2.202300223

**Published:** 2023-12-20

**Authors:** Yunis Khan, Roshan Raman, Zafar Said, Hakan Caliskan, Hiki Hong

**Affiliations:** ^1^ Department of Mechanical Engineering Delhi Technological University Delhi 110042 India; ^2^ Department of Multidisciplinary Engineering The NorthCap University Gurugram Haryana 122017 India; ^3^ Department of Sustainable and Renewable Energy Engineering University of Sharjah Sharjah 27272 UAE; ^4^ Department of Industrial and Mechanical Engineering Lebanese American University (LAU) Byblos 36 Lebanon; ^5^ Department of Mechanical Engineering Faculty of Engineering and Natural Sciences Usak University Usak 64200 Turkey; ^6^ Department of Mechanical Engineering Kyung Hee University Yongin 17104 Republic of Korea

**Keywords:** concentrated solar power, energy‐exergy‐exergoenvironmental evaluation, pre‐compression helium Brayton cycle, solar power tower, transcritical CO_2_ cycle

## Abstract

Solar power tower technology has strong potential among the other concentration solar power techniques for large power generation. Therefore, it is necessary to make a new and efficient power conversion system for utilizing the solar power tower system. In present research, a novel combined cycle is proposed to generate power for the application of the solar power tower. The pre‐compression configuration of the Brayton cycle is used as a topping cycle in which helium is taken as the working fluid. The transcritical CO_2_ cycle is used as bottoming cycle for using the waste heat. The proposed system is investigated based on exergy, energy, and exergoenvironmental point of view using computational technique engineering equation solver. Also, the parametric analysis is carried out to check the impact of the different variables on the system performance. It is concluded that the overall plant's optimized thermal and exergy efficiencies are obtained as 31.59% and 33.12%, respectively, at 800 °C optimum temperature of combined cycle and 850 W m^−2^ of direct normal irradiation and 2.278 of compressor pressure ratio. However, exergetic stability factor and exergoenvironmental impact index are observed as 0.5952 and 0.6801 respectively. The present proposed system performs better than the previous studies with fewer components.

## Graphical abstract

A novel combined cycle is proposed for the application of the solar power tower. Pre‐compression configuration of Brayton cycle is used as a topping cycle in which helium is taken as working fluid. Transcritical CO_2_ cycle is used as bottoming cycle for using the waste heat. The proposed system is investigated based on exergy, energy, and exergoenvironmental point of view.

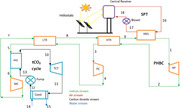

## 1 Introduction

Energy is essential to mankind, sustainability, and the advancement of technology. These days, the need for energy increases linearly with numerous factors, such as the expansion of the human population, advancements in technology, and industrial growth. For instance, it is projected that the global basic energy requirements by 2040 will range from 800 to 900 EJ.^[^ ^1^ ^]^ The requirement for safe and clean energy is impressively met by the concentration solar power (CSP) technology. Because of its high‐temperature generation and thus improved cycle efficiency, the CSP system is currently receiving a lot of attention in the power production industry.^[^ ^2^ ^]^ The Brayton cycle of supercritical carbon dioxide (sCO_2_) has a minimum pressure that is higher (≈7400 kPa) than any other gas Brayton cycle or steam Rankine cycle (SRC), making it a potentially useful technology for producing power from such a high‐temperature source.^[^ ^3^ ^]^ The sCO_2_ is a great substitute since it is non‐toxic, inexpensive, non‐corrosive, non‐flammable, and chemically stable. The working fluid in the supercritical Brayton cycle (SBC), SRC, and organic Rankine cycle (ORC) is pressurized by the compressor instead of a pump and stays supercritical throughout the SBC. These are the key differences between the three processes. Because CO_2_ has a high density, it is possible to create very light turbo‐machinery, which is attractive for applications involving the recovery of waste heat in automobiles. Due to the better performance of both supercritical and transcritical CO_2_ (tCO_2_) cycles in high‐temperature waste heat recovery increased attention nowadays.^[^ ^4^ ^]^ The supercritical and transcritical CO_2_ power cycles enhance the efficiency of heat exchangers and turbines. Thus, there has been a lot of interest in CO_2_ power cycles. For waste recovery, using this cycle in conjunction with an ORC or other bottoming cycle performed better than using a regular sCO_2_ cycle alone. These integrated cycles become less sensitive to the environment, more sustainable, and safer when powered by solar heat. In this regard, a SPT‐driven multigeneration system for heating, cooling, power generation, drying production, and hydrogen production was proposed by Yilmaz et al.^[^ ^4^ ^]^ .According to their findings of the sustainability and thermodynamic analyses, the suggested system's overall energy and exergetic efficiency were found to be 58.37% and 60.14%, respectively. With 18 775 kW, the solar tower sub‐system has the highest irreversibility of all the components in the multigeneration system. Yilmaz^[^ ^5^ ^]^ also suggested an additional SPT‐operated multigeneration system in a different study for flash desalination, combined cooling, heating, and power production. He came to the conclusion that the system's overall energy and exergy efficiency were determined to be 47.56% and 78.93%, respectively. Furthermore, the suggested system's fresh water and hydrogen productions are calculated to be 0.8862 and 0.04663 kg s^−1^, respectively. Khatoon and Kim^[^ ^6^ ^]^ investigated the recompression sCO_2_ cycle and tCO_2_ cycle when operated by a solar power tower (SPT) technique. It was claimed that a combined cycle was better than a single power cycle in terms of power conversion. Khan and Mishra^[^ ^7^ ^]^ studied solar‐driven partial heating cycle combined with ORC as a waste heat recovery cycle. It was concluded that combined cycle thermal efficiency obtained a value of 48.61% at 950 W m^−2^ of DNI.

In addition, Zare and Hasanzadeh^[^ ^8^ ^]^ suggested a combined helium Brayton cycle (HBC) with two ORCs run by SPT system in the direction of helium fluid. They were able to acquire a power plant's energy efficiency of above 30%. In an optimization study of the recompression cycle, Miao et al.^[^ ^9^ ^]^ used a mixture of nitrous oxide and helium as the working fluid rather than sCO_2_. It was found that, in comparison to N_2_O and CO_2_, the suggested method increased thermal efficiency by 5.1% and 6.5%, respectively. Under ideal circumstances, the optimized thermal efficiency was discovered to be 42.67%. The hydrogen liquefaction technique based on the HBC was introduced by Bi and Lu.^[^ ^10^ ^]^ It was concluded that the system under consideration could produce hydrogen and liquefy gas with less energy usage. Thermodynamic calculations of a closed Brayton cycle compressor using a helium/xenon combination as the working fluid were proposed by Malik et al.^[^ ^11^ ^]^ In a closed Brayton cycle, the number of stages in the compressor has been reduced from 16 to 3. Therefore, it is advantageous to use helium xenon rather than pure helium in power plant turbo‐compressors. In order to preserve food while employing the SPT system, Khan and Mishra^[^ ^12^ ^]^ recently suggested a recuperated HBC in conjunction with a cascading vapor compression‐absorption refrigeration system. The displayed plant's energy, exergy efficiency, and power output were determined to be 28.82%, 39.53%, and 14,865 kW, respectively. However, it was discovered that the heating and cooling COPs (coefficient of performance) were 1.539 and 0.5391, respectively. Khan et al.^[^ ^13^ ^]^ have suggested a recuperated HBC system in conjunction with a tCO_2_ cycle as a bottoming cycle to collect waste heat for the SPT system. They applied thermodynamic and exergoeconomic analyses to the proposed system. It was found that the SPT‐based combined cycle (SPT‐HBC‐tCO_2_ cycle) generates 34.68% energy efficiency at a cost of 1.613 cents per kWh for electricity. Zhou et al.^[^ ^14^ ^]^ designed the combined HBC combining ORC and vapor absorption refrigeration system for intake cooling of recuperated HBC system in order to optimally utilize the solar energy from SPT system. It was concluded that, in comparison to the basic HBC system, the optimized suggested combined cycle's exergy efficiency and electricity cost had improved by 14.5% and 11.9%, respectively.

The aforementioned literature analysis makes it clear that, despite the fact that HBCs are a fairly advanced technological advancement, their use is limited by their higher working temperature and that, because of their huge back‐work ratio, they are only effective at higher temperatures. When it comes to a Brayton cycle arrangement, helium is more economically advantageous than other working fluids.^[^ ^15^ ^]^ The main cause of the enhanced economic performance is the higher heat capacity of helium at higher temperatures, which lowers the mass flow rate of helium and, as a result, lowers component sizes and prices.^[^ ^16^ ^]^

There isn't much information in the literature review about the use of HBC in solar thermal facilities. Because different organic fluids have different favorable operating conditions, the ORC has historically been the most popular choice for low‐temperature applications. Because it has a better temperature‐matching glide in the evaporator over organic fluid, the tCO_2_ cycle offers a superior choice to recover heat from the high‐temperature heat source than the conventional ORC cycles. Issues with the evaporator's pinch‐point temperature arise when organic fluid is applied. The tCO_2_ performed better than ORC in terms of the comparable thermodynamic mean heat rejection temperature.^[^ ^17^ ^]^ The previous works were performed by Khan et al.^[^ ^13^ ^]^ where they considered recuperated HBC and tCO_2_ cycle and Khan et al.^[^ ^27^ ^]^ where they used combined pre‐compression sCO_2_ cycle and ORC and different SPT systems with molten salt as HTF. However, the present study deals with new configuration pre‐compression HBC and tCO_2_ combined cycle for SPT (air as HTF) utilization. As a result, the current work examines a novel system from the perspectives of thermodynamics and exergo‐environment that consists of a distinct configuration pre‐compression HBC (PHBC) and tCO_2_ cycle. The current study has found that SBC performance is greatly improved when helium fluid is used in the HBC's pre‐compression setup. In SBC systems, a considerable amount of thermal energy is lost to the atmosphere at temperatures between 150 and 250 °C in order to cool the working fluid at the compressor entry and lower compression power consumption. The tCO_2_ cycle is utilized as a bottoming cycle to recover waste heat to PHBC. Numerous studies^[^ ^18^, ^19^, ^20^ ^]^ have looked at how adaptable the tCO_2_ cycle is for recovering waste heat.

In light of the above deliberation, the current study's main objectives are:

1. To suggest a new efficient SBC system for helium working fluid SPT plants that uses the tCO_2_ cycle as the bottoming cycle to generate additional power.
2. Exergoenvironmental studies were carried out for the suggested pre‐compression configuration of HBC‐tCO_2_ combined cycle in order to undertake thermodynamic calculations.
3. To analyze the integrated suggested system parametrically in order to find the critical parameters.
4. To evaluate the suggested system's usefulness against earlier research studies.

## 2 System Description

The pre‐compressor is used in the basic Brayton cycle layout, which places it between the main turbine and main compressor, to create an outlet compressor pressure that is independent of the main compressor's inlet pressure. To prevent the pinch problem in this system, more heat exchange is needed than in a basic Brayton cycle.^[^ ^21^ ^]^ Furthermore, Khan and Mishra's work^[^ ^2^ ^]^ covered in full the pre‐compression configuration's value for power generation. Since the current SPT plant is built for peak load scenarios, energy storage is not required. The parametric study has covered the effects of solar energy and irradiation on the current SPT plant's performance in more detail.

**Figure 1:**
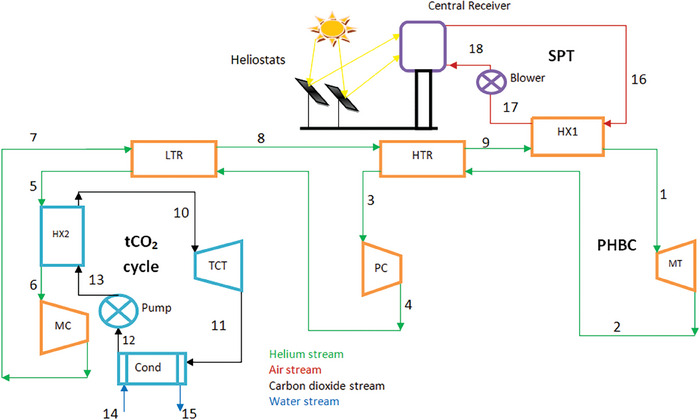
Schematic diagram of the proposed system.

In the current study, a unique combined cycle system (PHBC and tCO_2_ cycle) for harnessing the solar heat of the SPT system is developed. Three subsystems are depicted in **Figure** **1**: the waste heat recovery tCO_2_ cycle (bottoming cycle), the pre‐compression HBC (topping cycle), and the solar power tower subsystem. Air is regarded as a working fluid in the SPT system, which is used as a heat source. In this system, a volumetric air receiver is employed. The small alteration in the thermodynamic characteristics is caused by the blower in the SPT system.^[^ ^8^ ^]^ As a result, the diagram illustrates how close the thermodynamic characteristics are at stages 17 and 18. Heat exchanger 1 (HX1) transfers heat from the SPT to the topping pre‐compression HBC. In the topping cycle, helium is employed as the working fluid instead of supercritical CO_2_. The residual heat powers the waste heat recovery unit/heat exchanger 2 (HX2) and the bottoming tCO_2_ cycle. The working fluid flow path can be understood as follows: heated high‐temperature helium travels to the main turbine (MT) (states 1–2) for expansion after passing through HX1 (states 9–1) to absorb heat from the SPT system. The cold stream recovers its heat in the HTR (high‐temperature recuperator), which receives the expanded stream (states 2–3). The stream is then compressed once more in the pre‐compressor (PC) (states 3–4). The low temperature (LTR) (states 4–5) is traversed by the compressed stream, where heat is removed by the cold fluid. Heat exchanger 2 (HX2) (states 5–6) allows the tCO_2_ cycle to absorb the residual heat. Next, the main compressor (MC) (states 6–7) compresses the stream to the main turbine's input pressure. The HTR (states 8–9) travels to the HX1, whereas the LTR (states 7–8) is traversed by a chilly stream of helium.

In contrast to the topping cycle, the bottoming tCO_2_ cycle rejects some heat through the water as the heated stream of CO_2_ expands in the CO_2_ turbine (states 10–11), producing some shaft power. The heated stream of CO_2_ then travels to the condenser (Cond) (states 11–12), where some heat is rejected. After going through the pump (states 11–12), it enters HX2 once more to absorb heat (states 12–13). Thus, the circle comes back around.

## 3 Exergy‐Energy and Exergoenvironmental Analyses

During the examination of the current system, the following assumptions were made: 1) every component was operating in a steady state. 2) **Table** **1**’s pressure loss in the components is taken for granted. 3) Kinetic and potential energy were disregarded. 4) The compressor, pump, and turbine are assumed to have isentropic efficiencies, which are indicated in Table 1. 5) There are negligible changes in the thermodynamic properties due to the blower. For thermodynamic analysis, the entire system is split into the power cycle (PHBC‐tCO_2_ cycle) and the solar subsystem. The simulation model is created using Engineering Equation Solver (EES) and a thermodynamic model for each system component. The following is the formulation of the energy and exergy balance equations for a control volume in the context of a steady‐state process:

(1) $${\dot{Q}}_{CV}-{\dot{W}}_{CV}+\sum \left({\dot{m}}_{i}h_{i}\right)-\sum \left({\dot{m}}_{e}h_{e}\right)\;=\;0$$

(2)

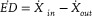

where, $\dot{\mathrm{ED}}$ denotes the rate at which exergy is destroyed within the component, ${\dot{X}}_{\mathrm{in}}$ and ${\dot{X}}_{\mathrm{out}}$stand for the total exergy rates that enter and exit the control volume, respectively. Excluding the energy resulting from height, velocity, and chemical exergy (because there is no chemical concentration in the suggested system), the rate of flow exergy of the fluid stream as the physical exergy can be expressed as follows.

(3) $${\dot{X}}_{j}={\dot{m}}^{•}\;\left[\left(h_{j}-h_{0}\right)-T_{0}\left(s_{j}-s_{0}\right)\right]$$

where ${\dot{X}}_{j}$ is the physical exergy at the jth state. The solar subsystem is composed of the receiver and the heliostat field. The former concentrates and reflects solar radiation energy onto the primary receiver using thousands of heliostats. The aperture area of every heliostat is *A*_hel_. The heliostats' receipt of solar heat has been reported by^[^ ^12^, ^13^ ^]^;

(4) $${\dot{Q}}_{Sun}=\;DNI\;\times A_{hel}\times N_{hel}$$

where, *N*_hel_ are the numbers of heliostats. The DNI varies according to the time of day and where you are on the earth. The remaining solar radiation, which is still useful heat for the receiver, has been lost to the environment to the extent that it was projected onto the heliostats. Consequently, the expression for heat directed onto the receiver is^[^ ^12^, ^13^ ^]^;

(5) $${\dot{Q}}_{rec,\;in}=\eta_{field}\;\times {\dot{Q}}_{Sun}$$

where, η_field_ is the heliostat field efficiency (optical), and expressed as^[^ ^22^ ^]^;

(6) $$\eta_{field}=\eta_{cos}\;\times \;\eta_{s\&b}\;\times \eta_{int}\times \eta_{att}\times \eta_{ref}$$

where,η_cos_,$\;\eta_{s\&b}$, η_int_, η_att_, η_ref_ represent the efficiencies of the cosine effect, shading and blocking, interception efficiency, atmospheric attenuation, and heliostats reflectivity, respectively. It is noteworthy that the current research considers the genuine values of an existing solar power plant, and the computation of these parameters is outside its scope. The ${\dot{Q}}_{\mathrm{rec},\;\mathrm{in}}$ represents the heat absorbed by the receiver, while ${\dot{Q}}_{\mathrm{rec},\;\mathrm{loss}}$represents the heat lost to the atmosphere as a result of conduction, convection, and reflection. The heat that remains ${\dot{Q}}_{\mathrm{rec},\;\mathrm{net}}$ is transferred to the heat transfer fluid, or air. Heat transport and receiver efficiency are represented as^[^ ^23^ ^]^;

(7) $$\eta_{rec}=\frac{{\dot{Q}}_{rec,\;net}}{{\dot{Q}}_{rec,\;in}}\;$$

(8) $${\dot{Q}}_{rec,\;in}={\dot{Q}}_{rec,\;net}\;+\;{\dot{Q}}_{rec,\;loss}={\dot{m}}_{air}\;\left(h_{16}-h_{18}\right)+{\dot{Q}}_{rec,\;loss}$$

**Table 1:** Simulation data of the proposed system.

Parameter	Value
Efficiency of heliostat field (η_field_)	0.6428^[^ ^8^ ^]^
Number of heliostats (N_hel_)	624^[^ ^8^ ^]^
Direct normal irradiation (DNI)	850 W m^−2[^ ^8^ ^]^
Reflective area of each heliostat (A_hel_)	9.45 × 12.84 m^2[^ ^8^ ^]^
Receiver aperture area (A_rec_)	68.1 m^2[^ ^8^ ^]^
Receiver efficiency (η_rec_)	0.75^[^ ^8^ ^]^
Temperature at the inlet of MT (T_1_)	800 °C^[^ ^8^ ^]^
Isentropic efficiency of main compressor (η_MC_)	0.89^[^ ^8^ ^]^
Effectiveness of heat exchanger (ε)	0.9^[^ ^8^ ^]^
Pressure at the inlet of MC (P_6_)	2500 kPa^[^ ^8^ ^]^
Isentropic efficiency of main turbine (η_MT_)	0.93^[^ ^14^ ^]^
Isentropic efficiency of tCO_2_ turbine (η_TCT_)	0.8^[^ ^13^ ^]^
Temperature at the inlet of tCO_2_ turbine (T_10_)	180 °C^[^ ^8^ ^]^
Pinch point difference in condenser	5 °C^[^ ^12^, ^13^ ^]^
Pinch point difference in HX2	10 °C^[^ ^8^ ^]^
Apparent temperature of the Sun (T_Sun_)	4500 K^[^ ^8^ ^]^
Atmospheric temperature (T_0_)	25 °C^[^ ^5^ ^]^
Atmospheric pressure (P_0_)	101.3 kPa^[^ ^5^ ^]^
Pressure loss in HX1	2%^[^ ^8^ ^]^
Pressure loss in HTR/HX2	1%^[^ ^8^ ^]^

The relationships that were used for the energy and exergy analyses of the system components are listed in **Table** **2**. Together with the input data specified in Table 2, these relationships are all incorporated into the simulation code, which is created using EES software. To find all of the unknown parameters, such as state point thermodynamic properties, heat and work interactions, and exergy rates for each stream, the application pulls the thermodynamic properties from its library of property functions. The total thermal and exergy efficiency for the studied solar power plant has been defined as the ratio of net output power to the energy or exergy input accessible with the sun irradiation on the heliostat field^[^ ^23^ ^]^:

(9) $$\eta_{th,\;overall}=\frac{{\dot{W}}_{net}}{{\dot{Q}}_{Sun}}\;$$

(10) $$\eta_{ex,\;overall}=\frac{{\dot{W}}_{net}}{{\dot{X}}_{Sun}}\;$$

**Table 2:** Equations for energy and exergy analyses of each component.

Component	Energy balance equation	Exergy balance equation
Heliostat field	$\;{\dot{Q}}_{rec,\;in}=\eta_{field}\;\times \;DNI\;\times A_{hel}\times N_{hel}$	${\dot{Q}}_{Sun}\times \;\left(1-\;\frac{T_{0}}{T_{ref,Sun}}\right)={\dot{Q}}_{rec,\;in}\;\times \left(1-\;\frac{T_{0}}{T_{ref,hel}}\right)+{\dot{ED}}_{hel}$
Receiver	$\;{\dot{Q}}_{rec,\;in}={\dot{m}}_{air}\;\left(h_{16}-h_{18}\right)+{\dot{Q}}_{rec,\;loss}$	${\dot{X}}_{18}+{\dot{Q}}_{rec,\;in}\times \;\left(1-\;\frac{T_{0}}{T_{ref,hel}}\right)={\dot{X}}_{16}\;+{\dot{Q}}_{rec,\;loss}\times \left(1-\;\frac{T_{0}}{T_{rec}}\right)+{\dot{ED}}_{rec}$
Heat exchanger 1	$\;{\dot{Q}}_{HX1}={\dot{m}}_{air}\;\times \left(h_{16}-h_{17}\right)+{\dot{m}}_{He}\times \left(h_{1}-h_{9}\right)$	${\dot{X}}_{16}-\;{\dot{X}}_{17}=\;{\dot{X}}_{1}-{\dot{X}}_{9}+{\dot{ED}}_{HX1}$
Main turbine	$\;{\dot{W}}_{MT}={\dot{m}}_{He}\;\times \left(h_{1}-h_{2}\right)$ $\;\eta_{MT}=\frac{\left(h_{1}-h_{2}\right)}{\left(h_{1}-h_{2s}\right)}\;$	$\;{\dot{X}}_{1}=\;{\dot{X}}_{2}+{\dot{W}}_{MT}+{\dot{ED}}_{MT}$
Main compressor	$\;{\dot{W}}_{MC}={\dot{m}}_{He}\;\times \left(h_{7}-h_{6}\right)$ $\;\eta_{MC}=\frac{\left(h_{7s}-h_{6}\right)}{\left(h_{7}-h_{6}\right)}\;$	$\;{\dot{X}}_{6}=\;{\dot{X}}_{7}-{\dot{W}}_{MC}+{\dot{ED}}_{MC}$
Pre‐compressor	$\;{\dot{W}}_{PC}={\dot{m}}_{He}\;\times \left(h_{4}-h_{3}\right)$ $\;\eta_{PC}=\frac{\left(h_{4s}-h_{3}\right)}{\left(h_{4}-h_{3}\right)}\;$	$\;{\dot{X}}_{3}=\;{\dot{X}}_{4}-{\dot{W}}_{PC}+{\dot{ED}}_{PC}$
High‐temperature recuperator	(*h*_9_ − *h*_8_) = (*h*_2_ − *h*_3_) $\;\varepsilon_{HTR}=\frac{\left(T_{9}-T_{8}\right)}{\left(T_{3}-T_{8}\right)}\;$	${\dot{X}}_{2}-\;{\dot{X}}_{3}=\;{\dot{X}}_{8}-{\dot{X}}_{9}+{\dot{ED}}_{HTR}$
Low‐temperature recuperator	(*h*_4_ − *h*_5_) = (*h*_8_ − *h*_7_) $\;\varepsilon_{HTR}=\frac{\left(T_{8}-T_{7}\right)}{\left(T_{4}-T_{7}\right)}\;$	${\dot{X}}_{4}-\;{\dot{X}}_{5}=\;{\dot{X}}_{8}-{\dot{X}}_{7}+{\dot{ED}}_{LTR}$
Heat exchanger 2	${\dot{m}}_{He}\times \;\left(h_{5}-h_{6}\right)={\dot{m}}_{CO2}\;\times \left(h_{10}-h_{13}\right)$	${\dot{X}}_{5}-\;{\dot{X}}_{6}=\;{\dot{X}}_{10}-{\dot{X}}_{13}+{\dot{ED}}_{HX2}$
Condenser	${\dot{m}}_{CO2}\times \left(h_{11}-h_{12}\right)={\dot{m}}_{water}\;\times \left(h_{15}-h_{14}\right)$	${\dot{X}}_{11}-\;{\dot{X}}_{12}=\;{\dot{X}}_{15}-{\dot{X}}_{14}+{\dot{ED}}_{Cond}$
tCO_2_ turbine	$\;{\dot{W}}_{TCT}={\dot{m}}_{CO2}\;\left(h_{10}-h_{11}\right)$ $\;\eta_{TCT}=\frac{\left(h_{10}-h_{11}\right)}{\left(h_{10}-h_{11s}\right)}\;$	$\;{\dot{X}}_{10}=\;{\dot{X}}_{11}+{\dot{W}}_{TCT}+{\dot{ED}}_{TCT}$
Pump	$\;{\dot{W}}_{Pump}={\dot{m}}_{CO2}\;\left(h_{13}-h_{12}\right)$ $\;\eta_{Pump}=\frac{\left(h_{13s}-h_{12}\right)}{\left(h_{13}-h_{12}\right)}\;$	$\;{\dot{X}}_{13}=\;{\dot{X}}_{12}-{\dot{W}}_{Pump}+{\dot{ED}}_{Pump}$

The exergy associated with solar irradiation or sun exergy can be calculated as;

(11) $${\dot{X}}_{Sun}={\dot{Q}}_{Sun}\;\times \left(1-\;\frac{T_{0}}{T_{ref,Sun}}\right)$$

where, *T*_ref,Sun_ is the apparent temperature (4500 K) of the sun that was used as the equivalent temperature of heat source for exergy analysis^[^ ^23^ ^]^ and ${\dot{W}}_{\mathrm{net}}$ is evaluated as:

(12) $${\dot{W}}_{net}={\dot{W}}_{MT}\;-{\dot{W}}_{MC}+{\dot{W}}_{TCT}-{\dot{W}}_{pump}$$

The exergy efficiency of each component is defined as the ratio of the useful exergy within the component to the total available exergy at the component. Mathematically, for jth component it can be calculated by;

(13) $$\eta_{ex,\;j}=\frac{{\dot{X}}_{useful}}{{\dot{X}}_{total,\;available}}\;$$

The combined cycle (PHBC‐tCO_2_ cycle) efficiency can be characterized for the power generation unit as a thermodynamic cycle that transforms the thermal energy absorbed in HX1 to mechanical power as follows^[^ ^13^, ^14^ ^]^:

(14) $$\eta_{th,\;combined\;cycle}=\frac{{\dot{W}}_{net}}{{\dot{Q}}_{HX1}}\;$$

(15) $$\eta_{ex,\;combined\;cycle}=\frac{{\dot{W}}_{net}}{\left({\dot{X}}_{16}-{\dot{X}}_{17}\right)}\;$$

where, $\left({\dot{X}}_{16}-{\dot{X}}_{17}\right)$ denotes available exergy input to the combined power cycle.

In addition to thermal analysis, it is now required to look into the sustainability of the power system by looking at how it affects the environment. Power plant thermodynamic assessments that only consider energy and exergy are insufficient. As a result, integrated multigenerational systems conduct environmental and sustainability analyses using exergy.^[^ ^24^ ^]^ The path of exergoenvironmental analysis is determined by the impact of exergy destruction rate and exergy efficiency on the surrounding environment. **Table** **3** lists the most important exergoenvironmental parameters. The exergoenvironmental impact factor has been taken to show how a thermal system affects the environment and how it can be utilized to reduce environmental hazards by minimizing irreversibilities. It was described as the ratio of total exergy destruction to the total available exergy to the system's inlet. The inverse of exergy efficiency is the exergoenvironmental impact coefficient, and its low value for the researched system is greatly praised. Whether a system is harmful to the environment or not is determined by the exergoenvironmental impact index. The lower the exergoenvironmental impact index value, the better the system's performance. Parameter exergoenvironmental impact improvement determines the system's relevance to environmental conditions.

**Table 3:** Exergoenvironmental evaluation.

Exergoenvironmental impact coefficient (*C_ei_*)^[^ ^24^ ^]^	*C_ei_* =$\frac{1}{\eta_{\mathrm{ex}\;}}$
Exergoenvironmental impact factor (*f_ei_*)	$\;f_{ei}=\frac{{\dot{ED}}_{total}}{{\dot{X}}_{solar}}\;$
Exergoenvironmental impact improvement (θ_*eii*_)	θ_*eii*_=$\frac{1}{\theta_{ei}}$
Exergy stability factor (*f_es_*)	$\;f_{es}=\frac{{\dot{X}}_{total,out}}{{\dot{ED}}_{total}+{\dot{X}}_{total,out}}\;$
Exergetic sustainability index (θ_*est*_)	θ_*est*_ = *f_es_* × θ_*eii*_
Exergoenvironmental impact index (θ_*ei*_)	θ_*ei*_ = *f_ei_* × *C_ei_*

In contrast to the exergoenvironmental effect index, a higher value of this parameter is thought to be better for the environment. The value of the energy stability factor should be close to “one.” A higher exergetic sustainability value is beneficial to the system. If it is lower, the system under investigation is harmful to the environment. The lower the value of the exergoenvironmental impact index is preferred for the better the system's performance. The exergoenvironmental analysis was conducted using modeling equations, and results were calculated using EES's computational techniques.

### 3.1 Optimization

Optimization minimizes or maximizes an objective function by changing several independent variables within a reasonable range. Single‐objective optimization is utilized when only one function is required; multi‐objective optimization is chosen when more than one objective is needed. The objective function of the power generation systems may be their efficacies, power, cost, and released pollutants.^[^ ^24^ ^]^ Numerous decision variables can influence the optimization's objective functions; as a result, more independent variables, also known as the degree of freedom, must be selected. Direct normal irradiation, turbine entry temperature, compressor intake temperature, and heat exchanger efficiency are all independent variables to take into account for system optimization. The primary performance criterion for every thermal power plant is thermal efficiency. As a result, the current single objective optimization issue considers thermal efficiency as its objective function; therefore, the current optimization's goal is to increase the system's thermal efficiency. The “optimization outcomes” section explains the optimization's methodology and process.

## 4 Validation of the Proposed System

The present model of the combined cycle is novel; no combined study is available in the literature. Therefore, this pre‐compression HBC and tCO_2_ cycle are validated separately. Thermal efficiency was considered as the parameter for the validation. Pre‐compression Brayton cycle and tCO_2_ cycle were validated with previous studies by Kim et al..[^[^ ^25^ ^]^] and Wang and Dai,^[^ ^26^ ^]^ respectively, at the same baseline conditions. The thermal efficiencies estimated errors were less than 1%, as shown in **Table** **4**. It shows that there is good agreement with the previous studies. It means that modeling equations for the considered system are authenticated. However, in the present study, instead of CO_2_ in the Brayton cycle, helium is used to analyze the central receiver concentration solar system.

**Table 4:** Validation of results.

Validation of pre‐compression Brayton cycle				
Working fluid	Base conditions	Thermal efficiency		Estimated error
		Kim et al..[^[^ ^25^ ^]^]	Current model	
CO_2_	MT inlet pressure = 27 180 kPa, MT inlet temperature = 384.85 °C, η_MT_ = 0.85, η_MC_ = 0.9	31.42%	31.6%	0.57%
Validation of bottoming tCO_2_ cycle				
CO_2_	Maximum pressure (*P*_10_) = 10 000 kPa, Minimum pressure (*P*_10_) Maximum temperature (T_10 _) = 65 °C, Minimum temperature (T_12_) = − 10 °C, η_TCT_ = 0.7, η_Pump _ = 0.8,	Wang and Dai^[^ ^26^ ^]^	Current model	0.04%
		8.484%	8.48%	0.01%

## 5 Results and Discussion

The basic facts and underlying assumptions are listed in Table 1. To give an overview of the energy and exergy assessment of the cycle under discussion, **Table** **5** displays the values of the study's findings for all subsystems at their optimal operating states. The entire power plant generated 16,431 kW of power from the total 64,358 kW of solar irradiation energy with an overall thermal efficiency of 25.39%, based on the data in Table 5, which is used in energy analysis. The heliostat field loses the majority of energy (22 989 kW, or 35.72% of the total solar energy), suggesting that the heliostat field's design is crucial to solar power tower systems. As can be observed, the combined cycle (PHBC‐tCO_2_ cycle) also has a pretty good thermal efficiency. Solar irradiance is a high‐quality energy source with a heat source that is at a high temperature of ≈4500 K^[^ ^8^, ^23^ ^]^ and has a significant irreversibility. The energy is received by the receiver at a temperature of ≈1000 °C.^[^ ^8^ ^]^ Exergy analysis shows that the heliostat field experiences the most increased exergy destruction. The heliostat's exergy efficiency was discovered to be 64.27%. There is no discernible temperature differential between the helium and HTF in the IHE since the combustion process, which is the main cause of irreversibility in conventional power systems, does not take place in the IHE. The combined cycle therefore has a high energy efficiency of roughly 72.97%. However, the power plant's overall exergy efficiency is rather low due to the significant exergy destructions in the receiver and heliostat field. Additionally, the component‐wise exergy characteristics were determined and provided in **Table** **6**. The primary turbine was found to have the highest exergy efficiency (97.25%) out of all the components. **Table** **7** lists, as numbered and shown in Figure 1, the values of pressure, temperature, enthalpy, mass flow rate, exergy rate, and entropy for the operating conditions assumed in Table 1.

**Table 5:** Obtained results from the thermodynamic analysis at the given operating conditions.

Subsystem^a^ ^)^	Energetic evaluation				Exergetic evaluation			
	Input [kW]	Output [kW]	Loss [kW]	Thermal efficiency	Input [kW]	Output [kW]	Destruction [kW]	Exergy efficiency
Heliostat field	64 358	41 369	22 989	64.27%	60 094	38 628	21 465	64.27%
Solar receiver	41 369	31 027	10 342	75%	38 628	22 515	16 113	58.28%
Combined cycle	31 027	16 341	15 786	52.66%	22 515	16 431	7586	72.97%
Overall power plant	64 358	16 341	49 117	25.39%	60 094	16 431	43 663	27.34%

^a)^T_1_ = 800 °C, T_10_ = 180 °C, DNI = 850 W m^−2^.

**Table 6:** Exergy destruction and exergy efficiency for each component.

Component	Exergy destruction [kW]	Exergy efficiency [%]
Heliostats	21 465.0	64.27
Receiver	16 113.0	58.28
Heat exchanger 1	1079.0	91.65
Main turbine	778.6	97.25
HTR	643.0	92.91
LTR	650.0	91.45
Heat exchanger	667.6	81.95
Main compressor	877.0	92.42
Pump	405.9	15.10
tCO_2_ turbine	424.9	82.69
Condenser	258.0	29.48
Pre‐compressor	301.0	86.68

**Table 7:** Thermodynamic properties and the mass flow rates of the proposed system.

State	Working Fluid	$\dot{m}$ [kg s^−1^]	P [kPa]	T [°C]	h [kJ kg^−1^ K]	s [kJ kg^−1^ K]	$\dot{EX}$ [kW]
1	Helium	19.58	5659	800	4040	−1.703	89 040
2	Helium	19.58	2577	530.70	2634	−1.569	60 727
3	Helium	19.58	2551	203.70	936.10	−4.259	43 190
4	Helium	19.58	2525	73.28	258.80	−5.898	39 492
5	Helium	19.58	2434	70.98	345.89	−7.897	51 344
6	Helium	19.58	2500	30	34.04	−6.569	39 014
7	Helium	19.58	5833	167.40	757.70	−6.386	52 110
8	Helium	19.58	5788	180.30	678.90	−6.879	15 452
9	Helium	19.58	5774	494.60	2455	−3.483	68 404
10	CO_2_	56.47	21 879	180	56.01	−0.782	16 353
11	CO_2_	56.47	7214	87.83	3.13	−0.745	12 743
12	CO_2_	56.47	7214	30	‐204	−1.401	12 092
13	CO_2_	56.47	21 879	63.28	‐178.80	−1.390	13 323
14	Water	279.60	101.3	25	104.80	0.366	0
15	Water	279.60	101.3	35	146.70	0.504	191.9
16	Air	46.20	101.3	1125	1513	7.359	33 205
17	Air	46.20	101.3	544.60	841.80	6.741	10 690
18	Air	46.20	101.3	544.62	841.81	6.742	10 690

A parametric evaluation has also been conducted to analyze the impacts of different variables on the power plant's performance. The variations have been assessed while keeping the other parameters, listed in Table 1, constant. The effects of these parameters are discussed individually throughout the remainder of this section.

### 5.1 Compressor Pressure Ratio Effects on Performance

**Figure 2:**
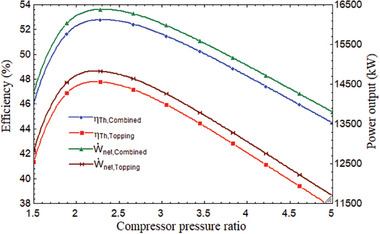
Performance comparison of topping and combined cycle with CPR.

Investigating the compressor pressure ratio (CPR) is a crucial variable. It had an impact on the material, or cost, of the compressor. **Figure** **2** illustrates how energy efficiency, or thermal efficiency, gradually declines after first increasing. At a CPR of 2.278, maximum thermal efficiency was discovered. At this stage, the stand‐alone and combined cycle's maximum thermal efficiencies were 47.78% and 52.79%, respectively. The bottoming cycle thermal efficiency increased by 9.49% when the tCO_2_ cycle was added to the stand‐alone (pre‐compression HBC), as shown in Figure 2. At 2.278 CPR, the power output for the combined and stand‐alone cycles was 16 378 and 14 824 kW, respectively. As a result, the power output improved by 9.48%. By fixing the turbine intake temperature at 800 °C, the tCO_2_ cycle inlet temperature at 180 °C, and the DNI at 850 W m^−2^ in accordance with Indian climate conditions, Figure 2 illustrates the variation in both cycle efficiencies.

Since the CPR improved thermal efficiency, the performance of the combined cycle (HBC‐tCO_2_ cycle) was investigated. First, the energy increased to a high of 2.3 CPR, after which it continued to decline. At 2.278 of CPR, the combined cycle's maximum thermal and exergy were discovered to be 52.79% and 72.66%, respectively. Following the 2.278 CPR, it started to decline. This pattern can be interpreted as it was prior to the CPR of 2.278 since the CPR increased both the expansion and the compression work; however, in this section, the rate of expansion work improvement outpaces the rate of compression work improvement. As a result, network output increased. As a result, the combined cycle's efficiency increases. Results were shown after the CPR of 2.278 and vice versa.

**Figure 3:**
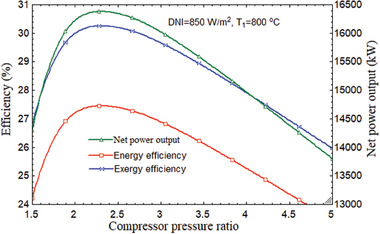
Performance variation of overall plant with CPR.

The variation of the thermal and exergy efficiency values with regard to the CPR are shown in **Figure** **3** in order to examine the exergy and energy losses in the solar subsystem. In the solar subsystem, a large portion of energy and force was lost or destroyed. The combination cycle's maximum values for thermal and energy efficiency were 52.79% and 72.66%, respectively, for the combined power cycle. By comparison, it was ≈27.45% and 30.25% for the entire power plant. The cycle may have lost a considerable amount of energy, but only a small amount of very low‐quality energy is lost, which explains the discrepancy between the cycle's high exergy efficiency and thermal and energy efficiency. Heliostats and the receiver in the sun field destroyed 86.06% of the total energy destruction. A total of 43 663 kW of plant energy destruction was discovered, with only 37 578 kW coming from damaged solar fields.

At 2.3 CPR, the combined cycle's maximum net power output was 16 378 kW. The inclusion of the tCO_2_ cycle to the stand‐alone HBC resulted in an increase in net maximum power output as compared to Figures 2 and 3. Maximum net power production for the combined cycle and the stand‐alone cycle were found as 16 378 and 14 824 kW, respectively, at 2.278 of CPR, which is the best value for CPR. Therefore, the tCO_2_ cycle, which serves as the bottoming cycle, enhanced the net power production by 1554 kW.

**Figure 4:**
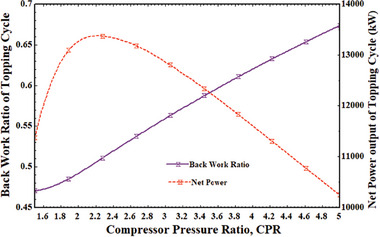
BWR and stand‐alone network output variation with CPR.

In addition to efficiencies as the system's performance metric, the back work ratio (BWR) is another important factor to consider. When CPR increases, BWR also increases. As demonstrated in **Figure** **4**, as CPR increased, the compression work rose steadily, leading to an increase in BWR. But after talking about how each other affects system performance, 2.278 is the most sensible figure for CPR. When the CPR increased from 1.5 to 5, the BWR climbed from 0.4712 to 0.6786.

**Figure 5:**
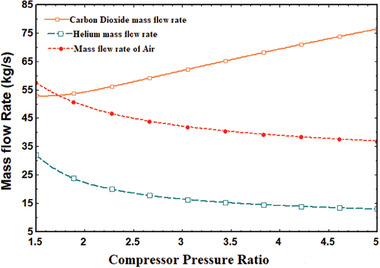
Mass flow rate variations with CPR.

The mass flow rates of the working fluids in several sub‐cycles, such as the air mass flow rate in the SPT system through the receiver, the helium mass flow rate in the topping cycle, and the carbon dioxide mass flow rate in the tCO_2_ cycle, were also impacted by the CPR. The mass flow rates of air and helium decreased with CPR while the mass flow rate of carbon dioxide increased, with all other input parameters remaining constant as previously discussed. Less helium flows down at the constant work output of the topping cycle as CPR rises because the pressure inside the topping cycle at the higher pressure side rises. For the same reason as previously mentioned, the air mass flow rate in the SPT system is now reduced along with the CPR. As CPR rose from 1.5 to 5, **Figure** **5** illustrates the variations in mass flow rates of air, helium, and carbon dioxide, which were, respectively, 57.45–37.56 kg s^−1^, 31.54–14.34 kg s^−1^, and 52.34–74.76 kg s^−1^.

### 5.2 Performance Variation with Pump Pressure Ratio

**Figure 6:**
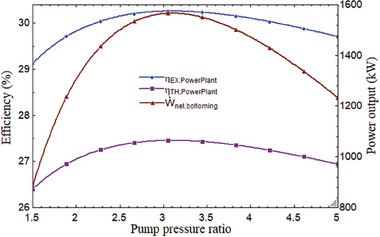
Overall plant performance and net power output of tCO_2_ cycle with PPR.

**Figure 7:**
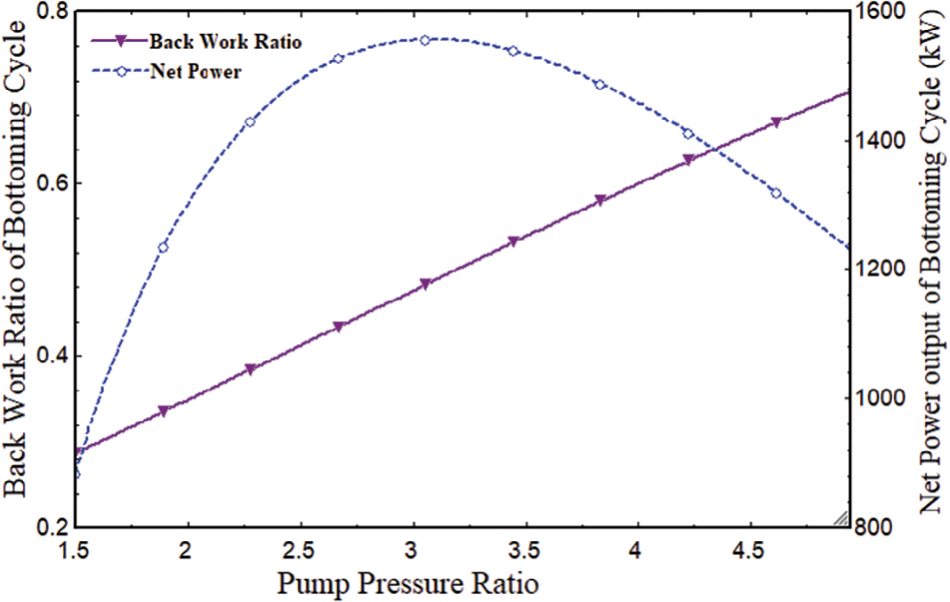
BWR variations with PPR.

The system's performance is affected by the pump pressure ratio (PPR). The thermal performance variation was determined at a fixed value of 800 °C for T_1_, 850 W m^−2^ for DNI, and 2.278 for CPR. The overall plant efficiency steadily declined after initially rising. It is at its best when the system is operating at its peak efficiency. As shown in **Figure** **6**, the greatest thermal and exergy efficiency of the entire power plant was attained at the optimal PPR of 3.056, which was 27.45% and 30.25%, respectively. The tCO_2_ cycle's net output power likewise grew initially before declining. It discovered a maximum at 3.056 PPR, which was the optimal value. Its maximum value was obtained as 1552 kW, the optimum value of PPR as illustrated in **Figure** **7**. The PPR also affects the combined cycle's BWR. BWR steadily increased as PPR did. Figure 7 shows that when PPR varied from 1.5 to 5, it grew from 0.3 to 0.72. This indicated that 3.03 PPR is the ideal value; at this time, the BWR is 0.49, indicating that 49% of the network's output is being utilized by the compressor.

### 5.3 Helium Turbine Inlet Temperature Effect

**Figure 8:**
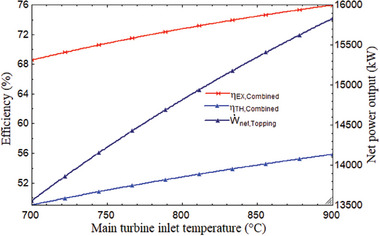
Efficiencies of the combined cycle and net power of topping cycle versus ITMT.

**Figure 9:**
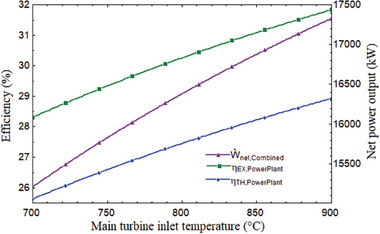
Performance variation of overall plant with MTIT.

**Figure 10:**
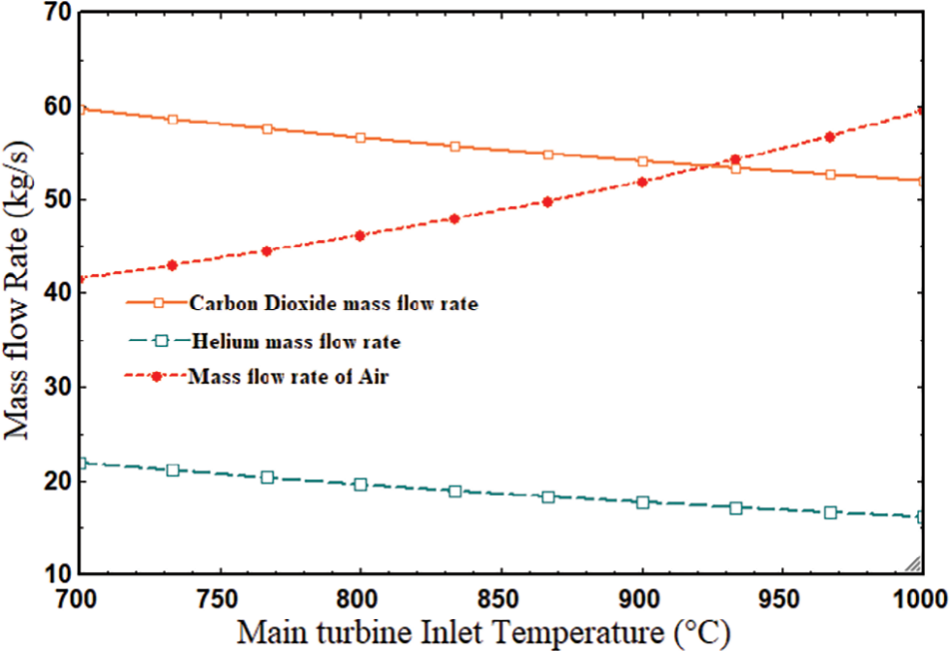
Mass flow rate variations with MTIT.

The performance of the system is influenced by the main turbine's inlet temperature (MTIT). With the MTIT, the combined cycle's efficiency and network output rose. The energy, thermal, and net power of the MTIT increased from 68.54% to 75.96% and from 49.02% to 55.85% when the temperature rose from 700 to 900 °C. **Figure** **8** shows that the topping cycle power output rose from 13 557 to 15 832 kW. When the MTIT climbed from 700 to 900 °C, as shown in **Figure** **9**, the plant's overall thermal, exergy efficiency, and net power output increased from 25.63% to 28.92%, 28.31% to 31.83%, and 15 209 to 17 327 kW, respectively. It can be explained by the fact that as the enthalpy difference across the turbine increased, so did the inlet temperature. It indicates that net expansion work increased, improving net output power and, as a result, the thermal performance of the system. This variance was examined at the optimal CPR of 2.278, 850 W m^−2^ of DNI, and 180 °C for the tCO_2_ cycle's intake temperature. In addition to net power output and efficiency, MTIT has an impact on the working fluid's mass flow rate in the power plant under consideration. **Figure** **10** illustrates how the mass flow rate of air in the SPT subsystem rose when MTIT decreased the mass flow rates of helium and carbon dioxide. As MTIT climbed from 700 to 900 °C, Figure 10 shows that the mass flow rates of air, helium, and carbon dioxide altered, going from 41.63 to 59.57 kg s^−1^, 21.92 to 16.14 kg s^−1^, and 59.69 to 52.02 kg s^−1^, respectively.

### 5.4 Effects on System Performance of Parameters of the Solar Subsystem

**Figure 11:**
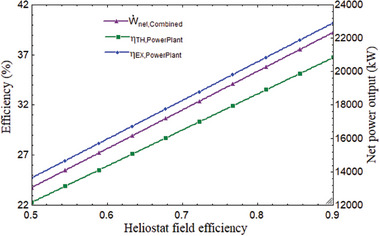
Variation of overall plant performance with heliostat field efficiency.

It is necessary to talk about the impact of SPT design factors in addition to the combined cycle parameters. The primary factor that has a greater impact on the power plant's performance is the heliostat field efficiency. The power plant's overall performance improved as the heliostat's efficiency rose. As demonstrated in **Figure** **11**, when heliostat efficiency increased from 0.5 to 0.9, exergy efficiency, power output, and thermal efficiency all increased from 24.74% to 40.1%, 22.3% to 36.74%, and 13 064 to 22 355 kW, respectively. This can be explained by an increase in heliostat efficiency, which reduced sun loss and boosted power conversion.

**Figure 12:**
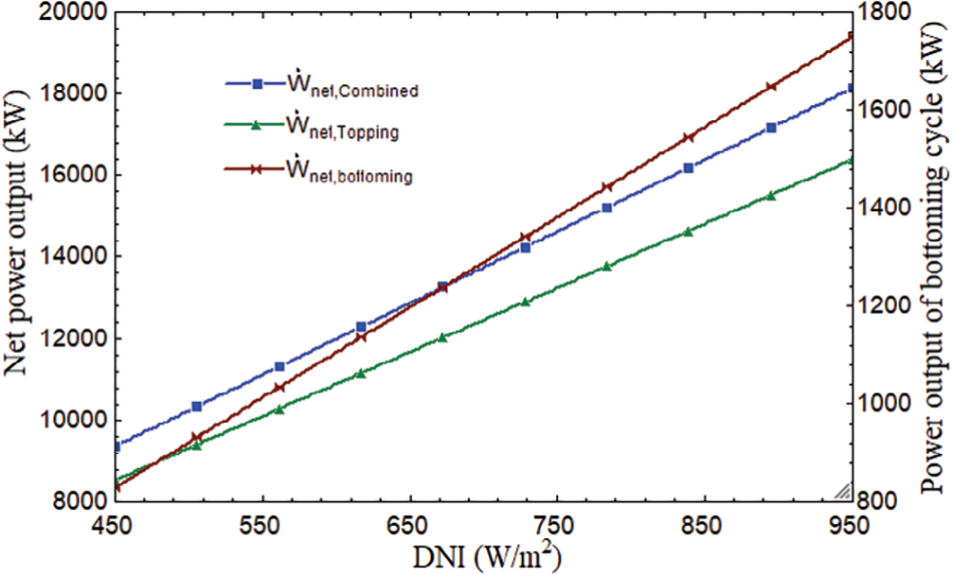
Power output variations with DNI.

As a result, output of power, exergy, and thermal efficiency increased. Moreover, power output rose as DNI did. The average DNI value for India's climate was 850 W m^−2^. The plant's thermal performance would improve as DNI grew since it would also boost receiver efficiency. As the DNI grew from 450 to 950 W m^−2^, the combined cycle's power output changed, ranging from 9355 to 18 138 kW, 8526 to 16 388 kW, and 828.7 to 1749 kW, respectively, as seen in **Figure** **12**.

### 5.5 Exergoenvironmental Performance Analysis

**Figure 13:**
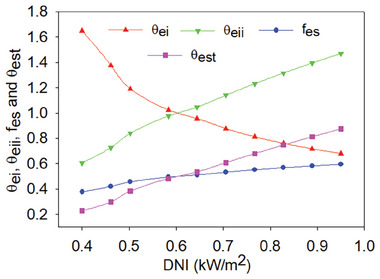
Exergoenvironmental parameters variation with DNI.

Exergoenvironmental evaluation, on the other hand, is used to examine the potential ecological repercussions of the proposed system. It is calculated how much energy is destroyed overall and how much energy correlates with each component. Then, one could decide whether or not the suggested system will negatively affect the environment based on this energy destruction. Exergoenvironmental parameters have thus been covered in this section. In addition, the exergoenvironmental impact index (θ_ei_) was examined to see whether the system under study was harmful to the environment. A lower score indicates that the system is better for the environment. With an increase in DNI, it lowers from 1.6504 to 0.6801 in our study, and its low value is environmentally friendly. The exergetic stability factor (*f*_es_) of “one” or moving in the direction of each indicates that the system is advantageous to the environment. **Figure** **13** shows that as DNI rises, this performance parameter increases from 0.3773 to 0.5952. The integrated energy efficiency increases to its maximum value as a result of the increase in solar intensity and the exergoenvironmental effect index (θ_ei_) zeroes out. Enhancement in exergoenvironmental impact (θ_eii_) will likewise arrive at infinite at the exact same time.

**Figure 14:**
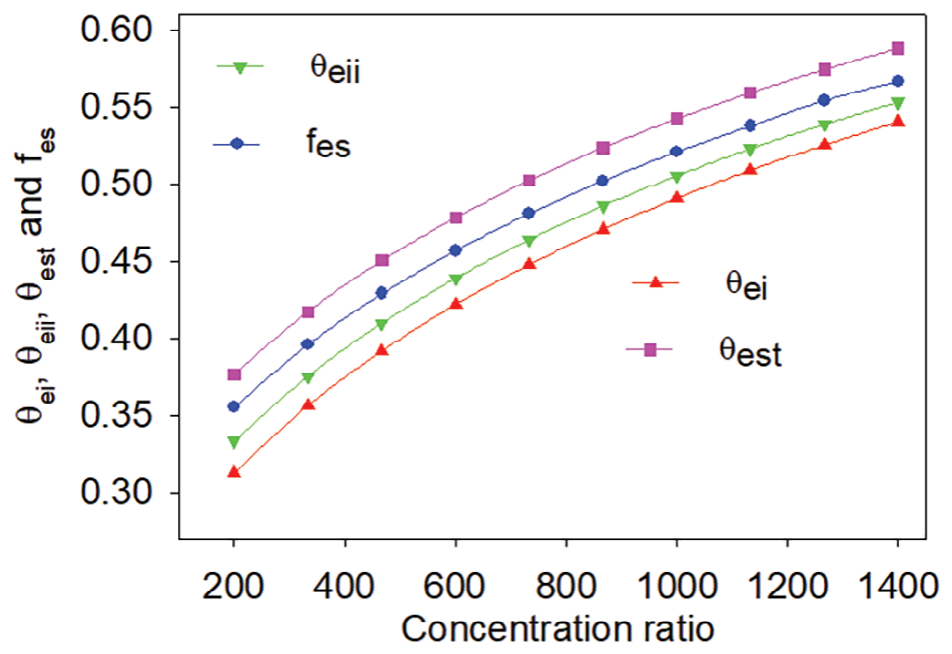
Exergoenvironmental parameters variation with concentration ratio.

**Figure 15:**
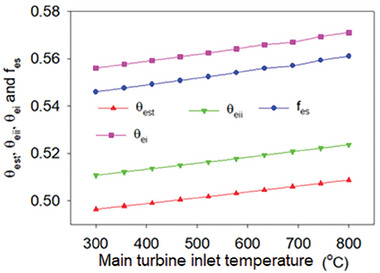
Exergoenvironmental parameters variation with MTIT.

As shown in **Figure** **14**, the exergoenvironmental impact index (θ_eii_), exergoenvironmental impact improvement (θ_eii_), exergy stability factor (*f*_es_), and exergetic sustainability index (θ_est_) all rose with the concentration ratio and reached values of 0.5403, 0.5530, 0.5666, and 0.5881 for the concentration ratio 1400. The heliostat area is proportional to the concentration ratio, increasing the quantity of heat that can be accessed from the Sun, which accounts for the enhancement in environmental performance as the concentration ratio rises. Additionally, it is discovered that accessible thermal gain is only greater than usable heat loss, improving the system's performance. In addition to the thermal performance, exergoenvironmental parameters such as the exergoenvironmental impact index (θ_ei_), exergoenvironmental impact improvement index (θ_eii_), exergy stability factor (*f*_es_), and exergetic sustainability index (θ_est_) increased with turbine inlet temperature and increased from 0.4965 to 0.5088, 0.5108 to 0.5237, 0.5460 to 0.5610, and 0.5560 to 0.5710, respectively, as shown in **Figure** **15**.

### 5.6 Optimization Results

Several parameters are optimized in this section of the research paper to achieve the best performance related to the best input value of the parameters. The EES tool's min/max property optimized the system. The objective function of the current two objective optimization issues was taken into consideration to be the power plant's thermal and exergy efficiency. **Table** **8** summarizes the various parameters together with their range, system bounds, and optimal values relative to the combined power plant's optimized efficiency. The main goal of this study is to increase the thermal and exergy efficiency of the integrated system, which is calculated in 4.5 s utilizing 550 equations, 131 iterations, and 201 blocks. According to earlier research by Yilmaz et al..[^[^ ^4^ ^]^] and Khatoon and Kim^[^ ^5^ ^]^ the DNI, turbine inlet temperature and heat exchanger efficiency were the three most important factors affecting the combined system's performance. In order to ascertain how these four factors affect the system's total thermal efficiency, they were chosen as independent variables along with their range.

**Table 8:** Optimum results of the present research.

Independent variables	Range	Optimum values
Heat exchanger effectiveness	0.8–0.95	0.95
Turbine inlet temperature (°C)	300–1200	850
DNI (kW m^−2^)	0.4–0.95	0.95
Compressor pressure ratio	1.5–5	2.278
Optimum thermal efficiency	–	31.59%
Optimum exergy efficiency	–	33.12%

### 5.7 Comparison with Previous Studies

The current system consists of a solar (SPT) system, PHBC, and a waste heat recovery tCO_2_ cycle as a bottoming cycle. This system has been compared with the previous research by Zare and Hasanzadeh.^[^ ^8^ ^]^ It can be seen that the current combined cycle (overall thermal efficiency 31.89%) is more thermally efficient than the Zare and Hasanzadeh^[^ ^8^ ^]^ (overall thermal efficiency 23.11%) with a minimum number of components. Zare and Hasanzadeh^[^ ^8^ ^]^ included 15 components, while the present study included only 10 components for greater thermal performance for high‐temperature solar power tower applications.

## 6 Conclusion

Present research deals with the energy‐exergy and exergoenvironmental analyses of the novel proposed combined cycle in which pre‐compression configuration of HBC is used as the topping cycle and tCO_2_ cycle as bottoming cycle to generate extra power for utilization of the solar power tower CSP system. Exergy and energy analyses revealed that it was beneficial to use the tCO_2_ cycle which improved the thermal efficiency by 9.48% of combined cycle as compared to the stand‐alone PHBC. Thermal and exergy efficiency of the combined cycle (PHBC‐tCO_2_ cycle) were found to be 52.79% and 72.66%, respectively, at 2.278 CPR, 850 W m^−2^ of DNI, and 800 °C of inlet temperature of the main turbine. However, thermal and exergy efficiency and net output power of the overall plant (SPT‐PHBC‐tCO_2_ cycle) were obtained as 27.45%, 30.25%, and 16 378 kW, respectively. It means that large a amount of exergy has been destructed in the SPT field (i.e., receiver and heliostats). Its values were ≈86.06% (37 578 kW) of total exergy destruction (43 663 kW) of the overall plant. Exergoeconomic analysis revealed that exergetic stability factor, exergoenvironmental impact index were observed as 0.5952 and 0.6801 respectively. However, the overall plant's optimized thermal and exergy efficiency was found to be 31.89% and 33.12%, respectively. The present proposed system performed better than the previous studies with fewer components. The cost analysis of the present study is the future scope of this research.

## Conflict of Interest

The authors declare no conflict of interest.

## Data Availability

The data that support the findings of this study are available from the corresponding author upon reasonable request.
